# Integration of DNA Damage and Repair with Murine Double-Minute 2 (Mdm2) in Tumorigenesis

**DOI:** 10.3390/ijms131216373

**Published:** 2012-12-03

**Authors:** Jason A. Lehman, Lindsey D. Mayo

**Affiliations:** Department of Pediatrics, Herman B Wells Center for Pediatrics Research, 1044 West Walnut Street, Indianapolis, IN 46202, USA; E-Mail: jlehman@iupui.edu

**Keywords:** base excision repair, cancer, homologous recombination, mismatch repair, murine double minute-2, non-homologous end joining, nucleotide excision repair

## Abstract

The alteration of tumorigenic pathways leading to cancer is a degenerative disease process typically involving inactivation of tumor suppressor proteins and hyperactivation of oncogenes. One such oncogenic protein product is the murine double-minute 2, or Mdm2. While, Mdm2 has been primarily associated as the negative regulator of the p53 tumor suppressor protein there are many p53-independent roles demonstrated for this oncogene. DNA damage and chemotherapeutic agents are known to activate Mdm2 and DNA repair pathways. There are five primary DNA repair pathways involved in the maintenance of genomic integrity: Nucleotide excision repair (NER), Base excision repair (BER), Mismatch repair (MMR), Non-homologous end joining (NHEJ) and homologous recombination (HR). In this review, we will briefly describe these pathways and also delineate the functional interaction of Mdm2 with multiple DNA repair proteins. We will illustrate the importance of these interactions with Mdm2 and discuss how this is important for tumor progression, cellular proliferation in cancer.

## 1. Introduction

### Human DNA Repair Pathways

Genotoxic stress can be induced through naturally occurring mechanisms, such as ultraviolet radiation and oxidative stress to individual DNA bases or it can occur through intentional DNA damaging agents in the case of chemotherapeutic anti-cancer agents. The multiple forms of genotoxic stress results in breaks within the DNA duplex or chemically altered DNA bases. Once genomic integrity is breached it then requires one of five primary human DNA repair pathways to fix breaks or replace bases. If repair is not initiated or faulty repair takes place and cell division is allowed to occur. This can lead to mutagenesis in key genes that ultimately initiates the development of cancer. Oncogenic pathways such as ones involving the murine double-minute 2 (Mdm2) have been shown to promote genomic instability. Mdm2 typically functions as an E3 ubiquitin ligase and conjugates ubiquitin to several proteins including, the tumor suppressor p53. This ultimately signals for destruction by the 26S proteasome to keep cellular p53 levels low [1,2]. p53 induces *mdm2* gene transcription at the conclusion of DNA repair to re-engage cellular homeostasis thus creating an autoregulatory feedback loop between Mdm2 and p53. However, under conditions of apoptotic cellular stress, p53 transactivates different subsets of target genes, such as *p21* for cell cycle arrest or *puma*, *bax*, *noxa* for apoptosis. Thus, a cell fate decision must be made to pause the cell in an arrested state and repair the DNA damage or if the extent of damage is too extensive then cellular death must be initiated. The complexity of maintain genomic integrity after DNA damage require, five primary DNA repair pathways: Base excision repair (BER), Homologous recombination (HR), Mismatch Repair (MMR), Nucleotide excision repair (NER) and Non-homologous end joining (NHEJ) or double-strand break repair. Each pathway will be briefly described illustrating the main protein and enzyme systems responsible for repair and how the oncoprotein Mdm2 may play a role in regulating these pathways.

## 2. Discussion

### 2.1. Base Excision Repair (BER)

BER attempts to fix single-strand breaks (SSBs), chemically altered bases and abasic sites. Base excision repair strives to reverse oxidative stress that occurs from reactive oxygen species (ROS) that occurs during normal metabolic events. 8-oxoguanine (8-oxoG) is one of the primary culprits for mutagenesis due to its ability to mimic a thymidine (T) DNA base thereby causing alterations in base pairing and bypassed by DNA replication polymerases [3]. After a damaged base is detected the first step is initiation of base removal through DNA glycosylases, such as NEIL1, NEIL2 and OGG1 [4–7]. DNA backbone cleavage is accomplished by AP lyase activity from DNA glycosylases or DNA AP endonuclease activity Removal of the *N*-glycosidic bond by these glycosylases generates an apurinic/apyrmidinic (AP) or a-basic site. The abasic lyase function of some of these DNA glycosylases possess the ability to generate 3′ unsaturated aldehyde within a SSB structure. The result of this leads to generation of single-strand breaks in an indirect mechanism, however a direct mechanism for single strand breaks can be generated through ROS. The direct mechanism utilizes poly (ADP) ribose polymerase-1 (PARP-1) and XRCC1 as recruitment and scaffolding complexes that bind to the abasic site [8]. The AP endonuclease (Ape1), carries out two distinct functions in generating a SSB: Formation of a 5′ dRP SSB due to hydrolysis of an abasic site or stimulation of the aforementioned DNA glycosylases to generate SSBs with 3′ modifications. Ape1 incises the DNA 5′ to the AP site, which forms a 5′ sugar residue that requires further processing by DNA polymerization and ligation.

Additionally, BER can be classified into short-patch BER for addition of one nucleotide and long-patch BER for 2–6 nucleotides. For resolution of the short-patch type repair after Ape1 activity is completed, polymerase β (pol β) activity removes the dRP group and replaces the nucleotide [9]. For the long-patch repair sub-pathway to be carried out it requires the activity of polymerase β, δ, ɛ, proliferating cell nuclear antigen (PCNA) for displacement of damage-containing strand and addition of multiple nucleotides. FLAP endonuclease-1 (FEN1) cleaves the damaged strand for removal. PARP-1 and high mobility group box 1 (HMGB1) also increase FEN1 cleavage activity [10,11]. The completion of BER occurs when the nicked strand is re-ligated independently by DNA ligase 1 or a combination of DNA ligase 3 and XRCC1 protein [12]. This represents a simplified view of the BER pathway and there are additional protein-binding partners and accessory proteins not mentioned here with key protein components summarized in Table 1. Thus, the BER protein pathways represent a fundamental protective mechanism in human cells against ROS, which could lead to mutation and eventually cancer.

Ref1/Ape1 represents one of the key enzymes in the BER pathway in which its importance is underscored by embryonic lethality and apoptosis in cells lacking this enzyme [13–15]. Ape1 was found to be polyubiquitinated in a human cancer cell line, HCT116, after H_2_O_2_ treatment and purified Ape1 protein mixed with HeLa cell extracts formed a distinct monoubiquitination pattern [16]. Examination of the E3 ligase Mdm2 showed that Mdm2 led to Ape1 monoubiquitination, while the RING-finger Mdm2 mutant (C464S) did not. Mdm2 and Ape1 were demonstrated to interact together under conditions of overexpression, yet not endogenous proteins. Monoubiqitination of Ape1 appears to also lead to exclusion from the nucleus, which the authors suggested might be a mechanism to be pro-apoptotic for interactions with Bcl-2 in mitochondria versus nuclear DNA repair [16]. In a subsequent paper the same group, used a T233E Ape1 mutant to look at how phosphorylation affects ubiquitination patterns [17]. This residue on Ape1 was shown to be a primary phosphorylation site for CDK5. A residue K48 when mutated to arginine reduced polyubiquitination on Ape1. Additionally, it was determined that when K24/K25/K27 residues were mutated the mono- and polyubiquitination patterns decreased in the T233E background [17]. These sites were at least partially dependent on Mdm2-mediated E3 ubiquitin ligase activity, but the authors acknowledged that Ape1 ubiquitination is controlled by additional ligases. Regulation of Ape1 through Mdm2 represents an intriguing mechanism of the base excision repair pathway. Considering that the formation of dRP site is the critical step of BER and Ape1 has additional roles in apoptosis, Mdm2 plays a pivotal role in this DNA repair pathway. It seems very plausible that Mdm2 could also regulate Ape1 indirectly by antagonizing protein binding partners such as XRCC1 or FEN1. This could be accomplished through non-E3 ubiquitin ligase mechanisms, such as binding by acidic domain of Mdm2, which was recently demonstrated to inhibit p53 transcriptional activity [18]. Another RING-finger independent function of Mdm2 has been demonstrated through the binding of Mdm2’s central domain, which caused a conformational change in p21/Waf1 leading to its alteration in half-life by the proteasome [19]. Thus, it appears that regulation of the BER protein, Ape1 by Mdm2 exhibits a typical ubiquitin ligase mechanism, which could become deregulated during various steps leading to establishment of a cancer cell.

### 2.2. Homologous Recombination (HR)

Homologous recombination repairs DNA double-strand breaks typically during mitotic late S-G2 cell cycle phases and utilizes a sister chromatid DNA as a repair template. HR does not utilize the error-prone repair from other pathways, such as Non-homologous end joining (NHEJ) which will be discussed in a later section, to promote genomic integrity. The key steps of HR are DNA end resection, strand invasion with a homologous sequence and DNA synthesis, addition of pre-synaptic complex on ssDNA, formation of Holliday junctions and resolution. The initial step after DNA damage is 5′–3′ end resection, which produces a 3′ single-stranded overhang that can invade a homologous template to prime repair [20]. The enzyme RAD51 has been shown to catalyze primary and multiple steps of HR Two other facilitators of DNA end resection, which promote Rad51-dependent strand exchange are human exonuclease 1 (*Exo1*) and the Bloom’s syndrome protein (BLM) [21]. The Mre11-Rad50-Nbs1 (MRN) nuclease complex manipulates double-strand break ends whereby the break ends are processed into 3′ overhangs of single-stranded DNA (ssDNA) [22,23]. Another early step involves the coating of ssDNA overhangs by replication protein A (RPA), a ssDNA binding protein. Formation of ssDNA-RAD51 nucleoprotein filaments cause strand invasion to search for homologous duplex DNA sequences as a stable intermediate [24]. While strand invasion occurs, formation of a displacement (D-loop) is carried out by RAD51 enzymatic activity and other cofactors. RAD51 D-loop formation is stimulated by replication protein A (RPA) and RAD52 [25–27]. DNA polymerases fill in 3′ overhangs, whereby the D-loop becomes a Holliday junction. RAD52 functions to anneal the second end of DNA leading to a Holliday junction. BLM migrates the two ends of the Holliday junction in the direction of one another with some filling in by polymerases if necessary. Completion of the Holliday junction structure is executed with DNA synthesis, ligation, branch migration and resolution. The Holliday junction DNA structure leads to either non-crossover or crossover products. Chromosomal crossover is determined by how the Holliday junctions are cut on both the crossing strand and the non-crossing DNA strand both leading to DNA structural resolution. Additional cofactors for homologous recombination utilizes the breast cancer susceptibility proteins 1 and 2 (BRCA1/BRCA2). BRCA2 is required for homology-directed repair in human cell lines dependent on interaction with RAD51 targeting it to ssDNA [28,29]. BRCA2 functions to promote RAD51 filamentation on DNA, in addition to site nucleation and stable filaments [30]. Of note, there are additional components not mentioned here which interact with BRAC1/BRAC2 and contribute to resolution of HR. A summary of key proteins involved in HR are summarized in Table 1. Therefore, the HR pathway functions to maintain chromosomal integrity especially during specific cell cycle phases and is a key component for prevention of cancer in human cells.

Mdm2 also has additional links to the homologous recombination repair pathway. One of the protein cofactors of the MRN complex involved in HR is Nbs1. Initially Mdm2 was shown to bind to the MRN complex in the absence of p53 through co-immunoprecipitation and mass spectrometry experiments [31]. This interaction was narrowed down to include only the Nbs1 protein, which was required for Mdm2 association with the MRN complex. Further, Mdm2 and Nbs1 were found to co-localize together at sites of DNA damage after cells were treated with ionizing radiation to induce double-strand breaks [31]. In a follow-up paper to this discovery, the authors looked at the effect of Mdm2 on genomic integrity in a p53-null background. Mdm2 overexpression led to an inhibition of Nbs1 directed repair of double strand breaks leading to elevated chromosome/chromatid breaks [32]. This also led to Mdm2-directed cellular transformation not dependent on p53. Mutation of a binding region critical for interaction with Mdm2 led to increased DSB repair rates. The fact that Mdm2 inhibits DSB repair and interacts with Nbs1 supports its role in oncogenesis. The authors point out that this unique interaction coupled with increased chromatid breaks due to increased Mdm2 protein levels can be one causative mechanism for p53-independent genomic instability and cellular transformation [33]. Maintenance of genomic integrity after DNA damage at the chromosomal level would be tightly regulated with a constrictive role being placed on Mdm2’s oncogenic function under normal conditions. It is intriguing that Mdm2 was found to specifically interact with Nbs1, since it is in complex with Mre11 and Rad50 as the MRN complex for chromosome break repair. Perhaps, Mdm2 might interact and likely regulate binding partners of Rad51 to antagonize HR mediated repair.

### 2.3. Mismatch Repair (MMR)

DNA mismatch repair (MMR) is an important DNA repair pathway, which facilitates removal of incorrect nucleotides on the opposite DNA strand. This typically stems from the error prone nature of DNA polymerases and misincorporation of the incorrect base and local dNTP pools [34]. Mismatched bases can be either a G/T or A/C pair. A recapitulation of human MMR was demonstrated in a cell-free system with the following minimal proteins: MutSα or MutSβ, Exonuclease 1 (*Exo1*), Replication factor C (RFC), proliferating cell nuclear antigen (PCNA), replication protein A (RPA), polymerase delta, and DNA ligase 1 [35–37]. To initiate MMR a nick in the DNA either 5′ or 3′ to the mismatch must occur. Proteins that bind the mismatch in humans are *E. coli* MutS homologues, which are MutSα and MutSβ [38]. MutSα is composed of Msh2/Msh6 heterodimer, while MutSβ is composed of Msh2/Msh3 proteins. MutSα is the first to bind the mismatch and exchanges ADP for ATP to become an active sliding clamp. MutSα recruits the MutL homologue MutLα, composed of a MLH1/PMS2 heterodimer. MutSα repairs single base substitutions and is capable of small loop repair, while MutSβ operates only in loop repair. The MutS protein complexes scan the duplex DNA and translocates along the DNA contour until it encounters proliferating cell nuclear antigen (PCNA) bound at the 3′ terminus of the nick. This multimeric complex recruits exonuclease 1 (*Exo1*) to incise the incorrect base via PCNA and replication factor C (RFC) [39]. Once the incorrect base is removed this allows a PCNA-polymerse ɛ complex to add the proper nucleotide. Key mismatch repair proteins are presented in Table 1. Therefore, correct Watson-Crick base pairing through mismatch repair is an essential repair process important for insurance that correct transcription of genes is allowed.

A human homologue of Drosophila lethal (2) denticleless protein, (L2DTL) was identified by mass spectrometry as a protein found to interact with proliferating cell nuclear antigen (PCNA) and cullin 4/DNA damage binding protein 1 (CUL4/DDB1) E3 ligase complexes [40]. This multimeric protein complex regulated p53 polyubiquitination through Mdm2 and individual components were found to interact with both proteins. Data revealed UV damage induced CUL4/DDB1 and PCNA leading to Mdm2 proteolysis. While suggestive, these components have yet to be assigned a function in the DNA mismatch repair pathway. It should be pointed out that PCNA also participates in the BER pathway, therefore the possibility exists that there is molecular crosstalk between this important sliding clamp protein and Mdm2 in two very different DNA repair pathways. While Mdm2 is regulated in part by PCNA, it has not been investigated in the context of the MMR pathway. The data suggest that Mdm2 has the potential to play a role in the MMR pathway yet studies to substantiate a role have yet to be described mechanistically.

### 2.4. Nucleotide Excision Repair (NER)

Nucleotide excision repair (NER) promotes repair of bulky helix-distorting DNA lesions, which typically are from chemotherapeutic agents like cisplatin or the 6-4 photoproducts from UV damage. NER can be further divided into two sub-pathways transcription-coupled repair (TCR), which involves preferential repair of the transcribed DNA strand and global genomic repair (GGR). The general mechanism of NER involves scanning and detection of DNA lesion, formation of a denaturation bubble, damaged strand incision, removal of the lesion-containing oligonucleotide, gap filling, and DNA ligation [41]. The recognition step of step is carried out by a xeroderma pigmentosum group C complex (XPC-HR23B-Cen2) [42]. Smaller DNA damage lesions can also be recognized by the DDB1 (XPD)/DDB2 (XPE) heterodimer. DDB forms a complex with other protein components to form an ubiquitin ligase complex that regulates XPC and XPE through polyubiquitination [43,44]. Transcription factor IIH (TFIIH) denatures duplex DNA to form a bubble approximately 30 nucleotides around the site of damage. This allows for greater accessibility in the unwound helix for NER protein complexes to bind. The replication protein A (RPA) and xeroderma pigmentosum group A (XPA) protein form an interaction together as part of the recognition step of bulky DNA adducts. This allows for exposure of the damaged single strand DNA. Incision of the damaged strand is enzymatically performed by endonucleases, XPG at the 3′ end in conjunction with XPF-ERCC1 at the 5′ end [45]. Gap filling is carried out by multiple DNA polymerases with some additional accessory proteins. The nucleotide excision repair protein complexes are in place to recognize and excise bulky DNA addicts typically caused from UV exposure and anti-cancer compounds summarized in Table 1.

One potential link between the nucleotide excision repair proteins and Mdm2 lies in a recent paper where the alternative reading frame (ARF) protein was demonstrated to stimulate XPC expression. ARF was found to be necessary for nucleotide excision repair in the absence of p53 [46]. Under most conditions ARF functions as a tumor suppressor by binding and inhibiting Mdm2 activity [47–49]. The authors used a series of cell lines that were either double knockout for p53/Mdm2^−/−^ or in some cases triple-knockout for ARF/p53/Mdm2^−/−^ and looked at repair of specific photoproducts and XPC levels. While they ruled out a p53-dependent ARF role, this data does not exclude an Mdm2-specific role, as Mdm2 is known to have many p53-independent functions and experiments were carried out in MEFs. These findings are suggestive that Mdm2 may serve a role in regulating XPC, perhaps through its ubiquitin ligase activity and warrants further investigation. In another really interesting paper from the same group they found that DDB2 is a cellular fate determinant after DNA damage. DDB2 was found to regulate between cell cycle arrest with DDB2 present versus cell cycle arrest when DDB2 is absent through experiments performed in WT and DDB2^−/−^ MEFs [50,51]. A pathway link between DDB2 and Mdm2 was established in experiments where Mdm2 inhibited apoptosis in conjunction with UV, cisplatin and doxorubicin in HeLa cells when DDB2 was silenced [50]. This effect was reversed with silencing Mdm2 in addition to DDB2 in the presence of DNA damage. Interestingly, they also found that the cell cycle arrest mediator p21^Waf1/Cip1^ is regulated by DDB2 to induce apoptosis. Whether DDB2 and Mdm2 form physical complexes with each other remains uninvestigated. Although, Mdm2 was not shown to act as an E3 ligase towards XPC and mediate 26S proteasomal degradation in these experiments, yet it certainly is not implausible. Future experiments could look at direct endogenous protein-protein interactions between XPC and its other protein-binding partners with Mdm2 and establish if overexpression leads to decreases in XPC levels. Regulation of the initial steps of NER through XPC and DDB2 would represent a novel function of Mdm2 in inhibition and suppression of global genomic NER.

It should also be pointed out as discussed in the homologous recombination section, that CUL4/DDB1 along with PCNA caused Mdm2 levels to be decreased in response to UV treatment [40]. Once again this illustrates crosstalk between Mdm2 and proteins that operate in more than a single DNA repair pathway. Y family type polymerases have been implicated in UV-induced DNA damage and also intimately linked to nucleotide excision repair. One such Y family member DNA polymerase, pol eta (pol H), is involved in global genomic-NER during S-phase [52]. Very recently, Mdm2 was found to associate with pol eta and turnover of this polymerase was targeted by Mdm2 when cells were treated with UV radiation [53]. Additionally, UV exposure to cells led to DNA pol eta polyubiquitination and proteasomal degradation by Mdm2. Thus, there are multiple nucleotide excision repair proteins, which are able to interact with Mdm2 and are subject to posttranslational modification by this E3 ubiquitin ligase.

### 2.5. Non-Homologous End Joining (NHEJ)

DNA double-strand break repair or non-homologous end joining (NHEJ) repairs breaks induced by ionizing radiation (IR), oxidative free radicals, misappropriate enzymatic action at sensitive sites, inhibition of topoisomerases, and other mechanical stresses [54]. Double-strand breaks (DSBs) within DNA represent a more difficult type of damage to repair and this can lead to more error-prone type of repair in some instances. NHEJ can function at any time during the cell cycle versus HR, which only operates during late S/G2 phases. NHEJ can be divided into four steps: recognition of DNA ends, synaptic complex formation, DNA end processing by nucleases, and ligation of DNA termini [55,56]. The very first protein to bind to the end of the double-strand break is the Ku70/Ku80 heterodimer. This unique protein forms a preformed channel so that it slides onto the DNA termini, causing a conformational change in Ku under both redox and DNA binding conditions [57,58].

Recruitment of the DNA-dependent protein kinase catalytic subunit (DNA-PKcs) is concomitant with binding to the *C*-terminal region of Ku80. Autophosphorylation of DNA-PK occurs when bound to specific sequence composition and DNA termini [59,60]. DNA-PK is believed to phosphorylate and activate downstream effectors [61]. The Artemis nuclease is responsible for end processing since blunt DNA ends are not always generated, especially after IR. Artemis utilizes a 5′ exonuclease and a 5′–3′ endonuclease to manipulate ssDNA overhangs for end processing [62,63]. Nucleotide addition is facilitated at these termini by multiple polymerases: β, μ, λ [64]. This entire process is regulated by protein-protein interactions such as the one formed by Ku/Ligase IV-XRCC4/polymerase μ [65]. The XRCC4 Like Factor (XLF/Cernunnos) functions biochemically to join with the XRCC4 protein to align DNA termini for more efficient end-joining [66,67]. DNA ligase IV completes the final steps of NHEJ to ligate DNA ends together while being held with XLF/XRCC4 and Ku/XRCC4 complexes [68,69]. Thus, the NHEJ protein machinery represents an important failsafe against lethal duplex-altering double-strand breaks. It is important to note that a recently discovered sub-pathway operates in conjunction with both NHEJ and HR called microhomology mediated end joining (MMEJ) utilizing a protein called CtIP [70,71]. This functions when DNA bases contain a small amount of sequence homology (microhomology), which can be either terminal on broken ends or embedded within the broken piece of DNA.

Once DNA repair factors have executed their enzymatic function they are no longer needed around duplex DNA. Interaction between Mdm2 and the non-homologous end joining proteins represents a potential avenue for exiting repair factors from repaired DNA. We previously identified a DNA-dependent kinase phosphorylation site on Mdm2 at Ser17 [72]. DNA-PK appears to function to negate the binding of Mdm2 to p53, which allows for cell cycle arrest and DNA repair to follow. P53 is then able to activate target genes for repair with Mdm2 taken out of the picture. Another NHEJ protein connection to Mdm2 in the literature is the Ku70 subunit, where these proteins were recently found to interact with each other. Mdm2 was determined to ubiquitinate Ku70 and decrease its levels in cells when Mdm2 is overexpressed or examined in *mdm2/p53*^−/−^ MEFs, yet not the RING-finger mutant C464S [73]. Interestingly, another tier of regulation was observed with Akt towards Ku70-Bax. Akt has been shown to phosphorylate Mdm2 on S166/S186 and allow Mdm2 to move from the cytoplasm to nuclear compartment [74]. Furthermore, Ku70 is able to bind Bax and inhibit its activity [75]. When Ku70 is ubiquitinated, Bax was observed to be active in response to DNA damage that promotes DSBs, and Akt inhibits Bax-mediated apoptosis partially through stabilization of Ku70 [73]. The authors hypothesize that Mdm2 mediated ubiquitination on Ku70 functions as a stress sensor and can be used as a regulatory step in apoptosis after DNA damage has ensued. Since Ku70 forms a pre-formed channel with the Ku80 subunit translocating down duplex DNA at sites of DSBs, removal of this protein after NHEJ is finished represents a unique mechanism. Removal of Ku could very well be accomplished post DSB repair by Mdm2 with some aspect of additional DNA-PK phosphorylation. Further dissection of NHEJ with Mdm2 and its binding partner, Mdmx warrants further investigation.

## 3. Conclusions

Accumulation of DNA damage and the inability to repair damage or repair gone awry directs human cells towards a tumorigenic phenotype, which is accompanied by cellular degeneration and finally cancer. Multiple DNA repair pathways regulate the fidelity of duplex DNA and are able to counteract specific subsets of lesions that can potentially alter genomic integrity. The murine double-minute 2 (Mdm2) oncogene functions as an E3 ubiquitin ligase towards some DNA repair enzymes and accessory proteins. The connection between Mdm2 and key proteins that mediate DNA repair has been understudied thus far. A summary of these DNA repair proteins direct and regulated interactions with Mdm2 is summarized in Table 2. During neoplastic development, disregulated DNA repair pathway play a role in genomic instability, missense mutations and chromosomal deletions and translocation. What remains unclear is how oncoproteins such as Mdm2 may play a role in preventing or promoting these genomic alterations as Mdm2 has been found in the nucleus during genotoxic stress. While the involvement of Mdm2 in DNA repair pathways are emerging, the integration of mouse models are beginning to be examined. Although, another complicating component is in cancer cells Mdm2 has many splice variants and the contribution of these variants to DNA repair is not known. Considering the development of small molecules to Mdm2, careful consideration must be taken Mdm2 with respect to these isoforms that lack the domains these small molecules bind. Thus, work on determining a role for Mdm2 in genotoxic stress is an understudied area and further work will determine its role in neoplastic development and resistance to common therapeutic modalities.

## Figures and Tables

**Table 1:** DNA repair pathways.

DNA repair pathway	Primary repair function	Major proteins involved in repair
Base Excision Repair (BER)	Alkylated, oxidized, deaminated bases, abasic sites	Ape1, DNA ligase 1/3, FEN1, HMGB1, NEIL1, NEIL2, OGG1, PARP-1, PCNA, Polymerase β, δ, ɛ, XRCC1
Homologous Recombination (HR)	Double strand breaks with strong sequence homology	BLM, BRCA1, Exo1, MRN, RAD51, RAD52, RPA,
Mismatch Repair (MMR)	Incorrectly paired bases on opposing DNA strands	DNA ligase 1, Exo1, MutSα (MSH2/MSH6), MutSβ (MSH2/MSH3), MutLα (MLH1/PMS2), PCNA, polymerase delta, RFC, RPA,
Non-Homologous End Joining (NHEJ)	Double strand breaks on blunt DNA ends or overhangs with little or no sequence homology	Artemis, DNA Ligase IV, DNA-PK Ku70/80, XLF, XRCC4, DNA Polymerases β, μ, λ
Nucleotide Excision Repair (NER)	Repairs bulky DNA adducts such as 6-4 photoproducts or cisplatin crosslinks	DDB1 (XPE), DDB2 (XBD), RPA, TFIIH, XPA, XPC, XPF-ERCC1, XPG, Y-type DNA polymerases

**Table 2:** Proteins in the DNA repair pathways that bind Mdm2.

DNA repair protein interaction/regulation with Mdm2	DNA repair pathway
Ape1	BER
NBS1	HR
Cul4/DDB1-PCNA	MMR
ARF/XPC	NER
Cul4/DDB1-PCNA	NER
DDB2 (XPD)	NER
Polymerase eta (pol H)	NER
DNA-PK	NHEJ
Ku70	NHEJ

## Abbreviations

AP: apurinic/apyrmidinic site
ARF: alternative reading frame
BER: base excision repair
BLM: Bloom’s Syndrome Protein
DNA-PK: DNA-dependent protein kinase
DSBs: double-strand breaks
GGR: global genomic repair
HR: homologous recombination
MMEJ: microhomology mediated end-joining
MMR: mismatch repair
MRN: Mre11-Rad50-Nbs1
Mdm2: murine double-minute 2
NHEJ: non-homologous end joining
NER: nucleotide excision repair
PARP: poly-ADP ribose polymerase
PCNA: proliferating cell nuclear antigen
RPA: replication protein A
ROS: reactive oxygen species
SSBs: single-strand breaks
TCR: transcription coupled repair
XPA: xeroderma pigmentosum group A protein
XPC: xeroderma pigmentosum group C protein
